# Simple and Low-Cost Oil/Water Separation Based on the Underwater Superoleophobicity of the Existing Materials in Our Life or Nature

**DOI:** 10.3389/fchem.2020.00507

**Published:** 2020-07-09

**Authors:** Hao Bian, Jiale Yong, Qing Yang, Xun Hou, Feng Chen

**Affiliations:** ^1^State Key Laboratory for Manufacturing System Engineering and Shaanxi Key Laboratory of Photonics Technology for Information, School of Electronics & Information Engineering, Xi'an Jiaotong University, Xi'an, China; ^2^School of Mechanical Engineering, Xi'an Jiaotong University, Xi'an, China

**Keywords:** oil/water separation, existing materials, underwater superoleophobicity, filter paper, zeolite layer

## Abstract

The achievement of high-efficiency oil/water separation has huge implications for protecting environment and reducing economic losses, but there is still a great challenge. Currently, most artificial oil/water separating materials are fabricated through complex preparation process, resulting in the very high cost of separation. In this paper, we present a simple and low-cost method to achieve oil/water separation by using the underwater superoleophobic materials that already exist in our life or nature. Taking filter paper and zeolite layer as examples, we show the inherent porous microstructures of these materials. Such porous microstructures endow filter paper and zeolite layer with strong ability to absorb water and the underwater superoleophobicity. Based on the porous feature and underwater superoleophobicity, the pre-wetted filter paper and zeolite layer can be used to effectively separate the mixture of water and oil, with great separation capacity. The existing materials (e.g., filter paper and zeolite layer) with both porous microstructure and underwater superoleophobicity in our life or nature are green and low-cost, and can be easily obtained. Such advantages allow those materials to potentially solve the pollution problems caused by the discharge of industrial oily wastewater and the oil-spill accidents.

## Introduction

Frequent oil spills severely threaten the marine ecosystems and the health of coastal inhabitants. For example, the famous Gulf of Mexico oil spill which occurred in 2010 released 200 million gallons of crude oil onto the sea. The discharge of industrial oily wastewater is continuously increasing year by year with the process of industrialization. In our daily life, the kitchen also produces a large amount of oil/water mixtures every day. The oil-spill accidents and the oily wastewater discharges not only cause huge economic losses but also lead to serious water pollution and other ecological and environmental problems (Xue et al., 2014; Chu et al., 2015; Wang et al., 2015a; Yong et al., 2016a, 2018b; Gupta et al., 2017). How to effectively separate the oil/water mixture and recycle the waster oil resources has become a worldwide concern. Although the traditional separating methods and materials are constantly improved in the practical applications, these oil/water separations still require a lot of manpower and financial resources, consume a great deal of separating materials, and cannot realize the recycle and reuse of the waste oils (Gupta et al., 2017). Recently, the porous materials with extreme wettability have attracted increasing interests due to their great potential in oil/water separation applications (Xue et al., 2014; Chu et al., 2015; Wang et al., 2015a; Yong et al., 2016b, 2018b; Gupta et al., 2017). Feng et al. (2004) prepared a stainless steel mesh that was coated with polytetrafluoroethylene micro/nanostructures. The mesh has both superhydrophobicity and superoleophilicity in air. Inverse wetting characteristics only allowed oil to pass through the mesh but intercepted water, thus achieving separation of water and oil. Xue et al. (2011) used a hydrogel-coated stainless steel mesh to separate the oil/water mixture. The as-prepared mesh showed superhydrophilicity in air while became superoleophobic after the immersion into water. When the mixture of water and oil was poured onto the mesh that was previously wetted by a little water, gravity driven the water phase to freely penetrate through the mesh and drop down, while the oil phase was repelled by the mesh and always maintained above the mesh because of the underwater superoleophobicity of the resultant mesh. Until now, more and more similar separating materials have been artificially fabricated and successfully applied in the field of oil/water separation (Zhang et al., 2013; Song et al., 2014, 2018; Kong et al., 2015; Liu et al., 2015; Cao et al., 2016; Yong et al., 2016b, 2019b; Yu et al., 2017; Wang et al., 2018; Yang et al., 2019). However, some deficiencies still hinder the large-scale practical application of these artificial separating materials. The fabrication of those separating materials usually requires the expensive original materials, the expensive equipments, or the complex preparation processes (Xue et al., 2014; Gupta et al., 2017). In the face of large-scale applications, these deficiencies will be infinitely amplified and become a huge burden. Thus, a simple, low-cost, green, and efficient method/material for separating oil/water mixtures is eagerly desired.

Here, we introduce the method of using the existing materials in our life or nature to effectively separate the mixture of water and oil. These materials have the features of inherent porous microstructure and underwater superoleophobicity. For example, the common filter paper and the layer of zeolite particles have natural microscale porous structure and micro/nanoscale hierarchical rough surface texture. Such materials have strong ability to absorb water in air (i.e., the superhydrophilicity) and repel oil droplets in water (i.e., the underwater superoleophobicity). The features of porous microstructure and the underwater superoleophobicity endow the filter paper and the zeolite layer with the great ability of oil/water separation. When the oil/water mixture was poured onto the pre-wetted filter paper or zeolite layer, only water could pass through these materials, while oil was maintained above the separating materials, achieving high-efficiency oil/water separation.

## Experimental Section

Filter paper and zeolite layer are selected as the examples to separate the mixture of water and oil. Such common materials already exist in our life or nature. In this experiment, filter paper and zeolite particles are purchased directly through Internet shopping. The zeolite particles are previously cleaned with water before measurement.

A simple oil/water separation device was designed based on the filter paper and zeolite particles. A plastic bottle (such as drink bottle) was removed its bottom half, and the rest top half was placed upside down. The bottle cap was drilled by a mechanical drill to form many holes with the diameter of 1–2 mm. A piece of metal mesh (300 mesh size) was placed inside the porous bottle cap as the support and also to prevent the separating materials from being lost. Then, the separating materials (the filter paper or the zeolite layer with the thickness of ~1 cm) were put onto the mesh, with tightening the cap. Before pouring the mixture of oil (petroleum ether) and water (V:V = 1:1) into the designed separating device, the filter paper and zeolite layer need be pre-wetted by a small amount of water. To distinguish oil and water, water was dyed by methylene blue and oil was dyed by Oil Red O.

The surface microstructures of the filter paper and zeolite layer were analyzed by a scanning electron microscope (Quantan 250 FEG, FEI, America). The static contact angle and dynamic sliding angle were measured via a contact-angle measurement (JC2000D, Powereach, China). Deionized water and 1,2-dichloroethane were used as the main detecting water and oil.

## Results and Discussion

**Figure 1:**
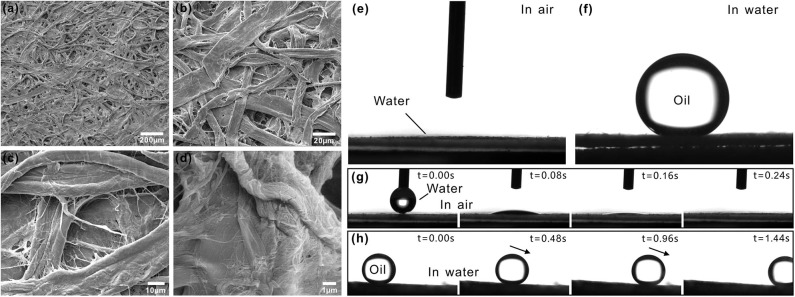
Surface morphology and wettability of a filter paper. **(a–d)** SEM images of the filter paper. **(e)** Water droplet on the filter paper in air. **(f)** Oil droplet on the filter paper in water. **(g)** Process of a small water droplet being absorbed by a filter paper. **(h)** Underwater oil droplet rolling on a filter paper.

Filter paper is a typical porous membrane in our life and is mainly made up of cotton fibers. Figures 1a–d shows the scanning electron microscopy (SEM) image of the surface microstructure of a filter paper. Fibers are mutually interlaced, resulting in lots of porous structures (Figures 1a,b). The diameter of these pores ranges from several to several ten microns. There are many nanoscale textures distributing on the surface of every fiber (Figures 1c,d). Therefore, the whole filter paper surface presents a kind of micro/nanoscale hierarchical porous structures. As a water drop was dripped onto the surface of the filter paper, the water drop would spread out quickly. Finally, the water drop would completely be absorbed by the paper. The measured contact angle (CA) of this water drop closed to 0°, so the filter paper showed superhydrophilicity in air (Figures 1e,g). When the filter paper was immersed in water, it was fully wetted. An underwater oil droplet that was placed on the surface of this filter paper could maintain a spherical shape, with the oil CA of 157 ± 2° (Figure 1f). In addition to such underwater superoleophobicity, the filter paper also showed extreme low adhesion to oil droplet in a water medium. Once the filter paper was tilted by 3 ± 1.5°, the underwater oil droplet would easily roll away (Figure 1h, Movie S1).

**Figure 2:**
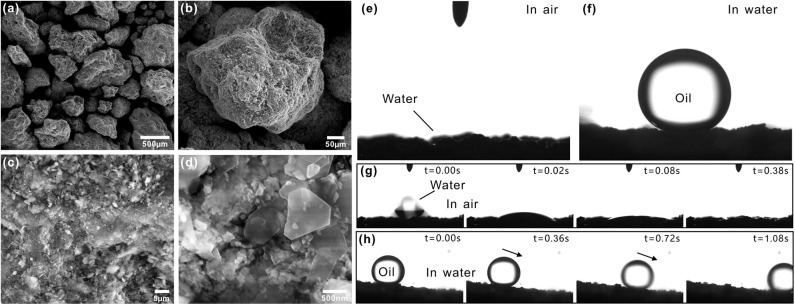
Surface morphology and wettability of the zeolite layer. **(a–d)** SEM images of the zeolite particles. **(e)** Water droplet on the zeolite particles in air. **(f)** Oil droplet on the zeolite particles in water. **(g)** Process of a small water droplet being absorbed by the layer of zeolite particles. **(h)** Underwater oil droplet rolling on the layer of zeolite particles.

Zeolite in nature is a kind of porous silicoaluminate mineral with water-containing cage structure. Its skeleton is SiO_4_ tetrahedral and AlO_4_ tetrahedral. Zeolite particles are able to stack up into material with rich gaps. Figures 2a–d depicts the microstructure of a layer of zeolite particles. The particles used in this experiment were 200–700 μm in diameter (Figure 2a). The zeolite layer was enrichment of pored structure formed between particles. The surfaces of the zeolite particles were not smooth but coated with rough micro/nanoscale hierarchical structure. There were abundant microscale pits and bumps on the zeolite particles (Figure 2b). The high-magnification SEM images revealed that no matter the surface of the pits or the bumps was further decorated with a large number of nanoscale block-like structures (Figures 2c,d). The cooperation between the hierarchical rough microstructure and the intrinsically hydrophilic chemical composition allows the zeolite layer to have the similar wettability to filter paper. The zeolite layer was superhydrophilic in air (Figure 2e). The water droplets were able to fully wet and finally permeate through the zeolite layer along the gaps between the zeolite particles (Figure 2g). By contrast, the pre-wetted zeolite layer exhibited underwater superoleophobicity and ultralow adhesion to oil droplets in water. The CA of an underwater oil droplet on the zeolite layer reached up to 154 ± 2.5° (Figure 2f) and the droplet could freely roll off on a 6 ± 2° tilted zeolite layer (Figure 2h, Movie S2).

**Figure 3:**
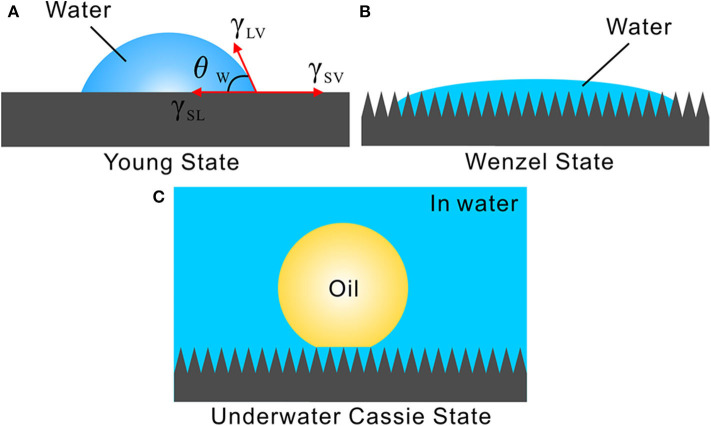
Different wetting states and the formation mechanism of underwater superoleophobicity. **(A)** Young wetting state. **(B)** Wenzel wetting state. **(C)** Underwater Cassie state for achieving underwater superoleophobicity.

Surface wettability is mainly governed by the chemical composition and surface morphology of a solid substrate (Genzer and Efimenko, 2006; Wang and Jiang, 2007; Wen et al., 2017; Yong et al., 2017a, 2018a, 2020; Bai et al., 2020; Wu et al., 2020). The inherent hydrophilic chemical composition and the micro/nanoscale hierarchical rough structures play an important role in the formation of the in-air superhydrophilicity and underwater superoleophobicity for the filter paper and the zeolite layer. These two substrates are made up of hydrophilic materials, with a large number of hydrophilic chemical groups decorating on their surfaces. Therefore, the filter paper and the zeolite are inherently hydrophilic substrates. The intrinsic wettability of a water droplet on a flat surface can be described by the Young's contact model, as shown in Figure 3A (Genzer and Efimenko, 2006). The Young's CA, θ_ω_, can be obtained from Equation (1):

(1) $$\begin{array}{l} \text{cos }\theta_{\omega }=\gamma_{SV}-\gamma_{SL}/\gamma_{LV} \end{array}$$

where the γ_*SV*_ is the surface energy of solid-vapor interface, the γ_*SL*_ is the surface energy of solid-liquid interface, and the γ_*SL*_ is the surface energy of liquid-vapor interface. Because of the high-surface-energy chemical compositions of the filter paper and the zeolite layer, it can be concluded that the θ_ω_ < 90°.

Wenzel pointed out that the rough surface microstructure also has great influence on the wettability of the materials besides their chemical composition. In the Wenzel wetting state, the liquid droplet wets the rough microstructures of the substrate surface so that the valleys of the microstructures are fully filled with liquid (Figure 3B) (Wang and Jiang, 2007; Wen et al., 2017). Taking into account the surface roughness, he carried on a modification to Young's equation as:

(2) $$\begin{array}{l} \text{cos}{\theta^{*}}_{\omega }=r(\gamma_{SV}-\gamma_{SL})/\gamma_{LV}=r\text{cos}\theta_{\omega } \end{array}$$

where θ^*^_ω_ and θ_ω_ are the apparent CA (water droplet on a rough substrate) and the Young's CA, and *r* is the roughness factor (the ratio of the actual surface area to the projected area). According to the Wenzel equation, hierarchical rough microstructures increase the real surface area of these materials, so the hydrophilicity is enhanced to superhydrophilicity (Wenzel, 1936; Wang et al., 2015b).

Once the samples are submerged in water, they will be fully wetted by water. Water can fill in the pores of the filter paper, the gap between the zeolite particles, and the surface microstructures of the samples, forming a trapped water layer. When an underwater oil droplet is placed on the sample surfaces, the wetting between the oil droplet and the filter paper/zeolite layer agrees well with the underwater version of the Cassie state (Figure 3C) (Cassie and Baxter, 1994; Liu et al., 2009; Yong et al., 2014, 2017b, 2019a). The trapped water layer provides the repellent force against oil droplets, making the filter paper and zeolite layer show underwater superoleophobicity.

**Figure 4:**
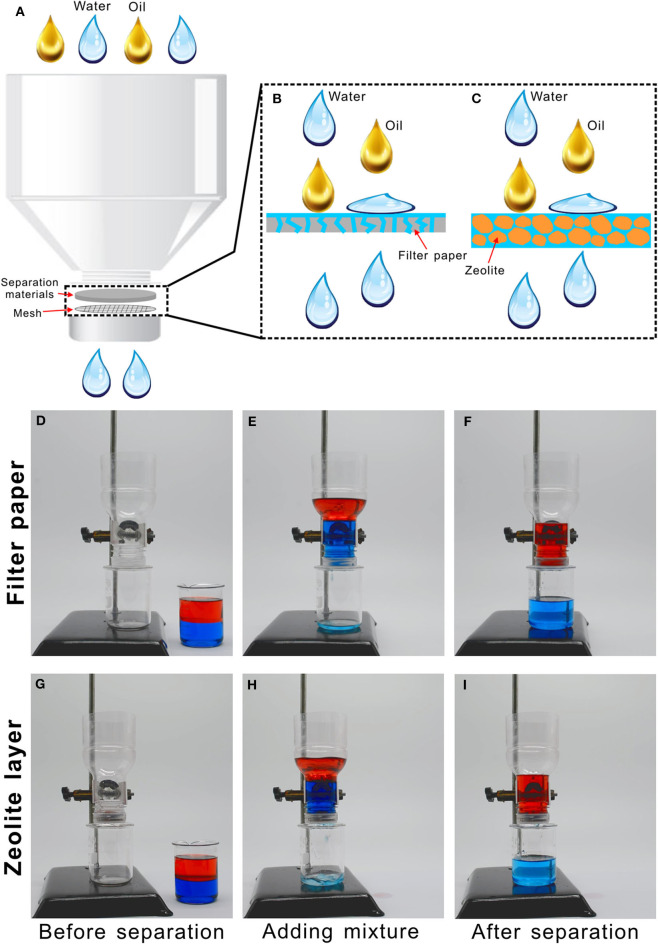
Oil/water separation by using the filter paper or zeolite layer as the separating materials. **(A)** Schematic of the separation setup. **(B,C)** Separating mechanism based on the pre-wetted **(B)** filter paper or **(C)** zeolite layer. **(D–I)** Process of oil/water separation by using the pre-wetted **(D–F)** filter paper or **(G–I)** zeolite layer. Water was dyed by methylene blue and oil was dyed by Oil Red O.

The features of porous microstructure and underwater superoleophobicity allow the filter paper and zeolite particles to be used to separate the oil/water mixture. By using the filter paper and zeolite layer as the separating materials, respectively, we designed a simple oil/water separation device. As shown in Figure 4A, a plastic bottle (such as drink bottle) was removed its bottom half, and the rest top half was placed upside down. The bottle cap was drilled by a mechanical drill to form many holes with the diameter of 1–2 mm. A piece of metal mesh (300 mesh size) was placed inside the porous bottle cap as the support and also to prevent the separating materials from being lost. Then, the separating materials (the filter paper or the zeolite layer with the thickness of ~1 cm) were put onto the mesh, with tightening the cap.

Figures 4D–F and Movie S3 shows the process of separating the mixture of water and oil by using filter paper. Before separation, the filter paper was wetted with a little volume of water. When the oil/water mixture was poured into the separating device, the water in the mixture could gradually penetrate through the filter paper and finally dripped into the below collecting beaker (Figure 4E). On the contrary, the oil phase always stayed above the filter paper, which was intercepted by the pre-wetted paper (Figure 4F). As all of the water passed through the filter paper, the separating process stopped. By this way, the oil/water mixture was successfully separated. Similarly, the pre-wetted zeolite layer also could separate the oil/water mixture based on the above-mentioned method and process (Figures 4G–I, Movie S4). The zeolite also exhibited excellent separating ability. If a new oil/water mixture was further added into the separating device, the stopped separating process would restart (Movie S5). The separation process can repeat at last 10 cycles. Therefore, such device can achieve repeated and continuous separation. The separation efficiency can be calculated by η = m_1_/m_0_, where m_1_ and m_0_ are the mass of the collected oil and the oil before separation. The measured separation efficiency for the filter paper was 96.02% and that for the zeolite layer was 97.23%. The collected oils can be recycled into use. The mechanism of separating the mixture of water and oil was schematically illustrated in Figures 4B,C. When the filter paper or the zeolite layer is previously wetted by water, water is able to enter into and fill in the microholes of the filter paper or the gaps between the zeolite particles. When the oil/water mixture is poured onto the pre-wetted separating materials, the superhydrophilicity of the separating materials ensures that the water phase in the mixture is easily absorbed by the materials and penetrates through the separating materials along the microholes of the filter paper (Figure 4B) or the gaps between the zeolite particles (Figure 4C). On the contrary, the underwater superoleophobicity endows the pre-wetted separating materials with the ability of repealing oil phase in the mixture, preventing the oil from passing through the filter paper or the zeolite layer. As a result, the mixture of oil and water is separated. Such treatment of the oil/water mixture not only avoids the environmental pollution but also save resources and energy.

Filter paper and zeolite particles have representative character and university. In the aspect of source, filter paper is from our life, while zeolite particles are from nature. Regarding the morphology, filter paper is a natural porous membrane, while zeolite particles are able to stack up into zeolite layer with rich gaps. The filter paper has good flexibility and can be folded into different shapes, such as box and cylinder. The filter paper is easy to carry and can be quickly assembled into separation device. Therefore, filter paper can be used to rapidly address the complex pollutions in emergency. The zeolite layer has good mechanical property and can be stack up into large equipment. For example, the zeolite particles can be piled up as a U-shaped channel, endowing the zeolite layer with the ability to separate a large amount of oil/water mixtures. Similar to filter paper and zeolite layer, there are a mass of existing materials in our life or in nature having both inherent porous microstructures and underwater superoleophobicity. Those materials can be potentially applied to separate the mixture of water and oil; that is, abundant natural separating materials for oil/water mixture actually exist in our life or in nature.

## Conclusions

In conclusion, we demonstrate that some existing materials (e.g., the common filter paper and the layer of zeolite particles) in our life or in nature have inherent microscale porous structure and micro/nanoscale hierarchical rough surface texture. By combining with the inherent hydrophilic chemical composition, these materials showed strong water-absorption ability as well as the superhydrophilicity in air, without any further treatment. When the filter paper and the zeolite layer were dipped into water and an oil droplet was put on their surfaces, the oil droplet was at the underwater Cassie wetting state, resulting in the underwater superoleophobicity of these porous materials. The ingenious combination of porous feature and underwater superoleophobicity endows the filter paper and the zeolite layer with the great ability of oil/water separation. When the mixture of water and oil was poured onto the pre-wetted filter paper or zeolite layer, only water was able to penetrate through these materials, while oil was maintained above the separating materials. Therefore, the mixture was successfully separated with high efficiency. The measured separation efficiency for the filter paper was 96.02% and that for the zeolite layer was 97.23%. Similar to the filter paper and zeolite layer, there are a mass of existing materials in our life or in nature having both inherent porous microstructures and underwater superoleophobicity, which can be potentially used as the separating materials for oil/water mixtures. Such existing materials are natural, green, and low-cost materials, and can be easily obtained, showing great advantages in solving the pollution problems caused by the discharge of industrial oily wastewater and the oil-spill accidents.

## Data Availability Statement

The original contributions presented in the study are included in the article/Supplementary Material, further inquiries can be directed to the corresponding author/s.

## Author Contributions

HB and JY designed the experiments and wrote the manuscript. FC directed and supervised the research. QY and XH contributed toward significant discussions and revised the paper.

## Conflict of Interest

The authors declare that the research was conducted in the absence of any commercial or financial relationships that could be construed as a potential conflict of interest.

## Supplementary Material

Movie S1 Process of an underwater oil droplet rolling on a filter paper.

Movie S2 Process of an underwater oil droplet rolling on the layer of zeolite particles.

Movie S3 Process of separating the mixture of water and oil by using the pre-wetted filter paper.

Movie S4 Process of separating the mixture of water and oil by using the pre-wetted zeolite layer.

Movie S5 Restarting the separating process after finishing a cycle of separation.
